# Air Pollution and Forest Health: Establishing Cause and Effect in the Forest

**DOI:** 10.1100/tsw.2001.73

**Published:** 2001-08-17

**Authors:** William J. Manning

**Affiliations:** University of Massachusetts, Amherst, MA, USA

**Keywords:** tree declines, metals, nitrogen, ozone, sulfur

## Abstract

I participated in a NATO Advanced Research Workshop titled “Effects of Air Pollution on Forest Health and Biodiversity in Forests of the Carpathian Mountains,” in Stara Lesna, Slovakia from May 22–26, 2001. Researchers from Canada, Czech Republic, Poland, Romania, Slovakia, Ukraine, and the U.S. met to present their results from a three-year cooperative study of tree health and air quality monitoring in forests of the Carpathian Mountains in Central Europe. Much of the work reported related to assessing the crown condition of trees in permanent plots in natural or managed (planted) forests in the mountains. The endpoint was tree condition, with results extrapolated to the forests in the Carpathian range. From this I learned that, of the 50,000 trees evaluated, European beech (Fagus sylvatica) was the most healthy, while Norway spruce (Picea abies) (the principal forest tree) and white fir (Abies alba) sustained crown defoliation of up to 12.8%. The cause of this crown defoliation and tree decline was usually attributed to “air pollution” as a generic term and an automatic assumption. It is well known that deposition of heavy metals and acidic sulfur and nitrogen compounds can cause tree decline and predispose affected trees to bark beetles and climatic damage. Chemical analyses can also be done to detect metals and sulfur compounds in trees and soils. Sometimes these analyses were done, but most often the assumption was that crown defoliation was caused by air pollution. The assumption was that given sufficient exposure to high enough concentrations of toxic elements, sooner or later there will be a visible adverse response.

The deposition of metals and sulfur compounds, but not nitrogen, to forests in Central Europe is declining. As former communist countries have become more affluent, the number of vehicles has expanded dramatically and combustion from all sources has also increased. These countries are also on the receiving end of long-range transport via continental winds from affluent countries such as Germany and Austria. Increasing levels of nitrogen dioxide ($NO_2$) from combustion and accompanying partially burned hydrocarbons have resulted in levels of ozone ($O_3$) that are above background and can be phytotoxic. The phytotoxicology of ozone is different than for metals and acidic compounds. As ozone is taken up by open stomata, a process driven by evapotranspiration, the assumption that a dose/response curve can be generated where exposure equals response is not the case. Ozone will be taken up if there is enough soil moisture and sunlight for stomata to function. There is also considerable genetic resistance to ozone in plant populations.

Ozone was measured in the Carpathians with a few active monitors and many passive samplers. GIS was used to depict the sampling points and indicate areas where ozone concentrations seemed high. The maps are extremely elegant, but indicate only where there is potential for ozone injury. My own work in the region has shown ozone injury symptoms on a variety of herbs and shrubs, but no symptoms on major forest trees.

Complicating the assessment of forest health is the genetic status of the forests. Most are planted with genetically uniform seedlings, following clear-cutting. There are not many natural forests. Several people at the meeting presented results that indicated that genetic diversity in Norway spruce was less in managed than in natural forests, making them more susceptible to various stressors.

Forests are complicated systems on which are superimposed air pollutants of various kinds, including a more recent threat, elevated concentrations of ozone. Given all the factors that affect forest function and health, it is extremely difficult to attribute cause and effect in the forest to air pollutants. Using tree condition as an endpoint will only tell us that a combination of factors has resulted in phytotoxic effects. Kevin Percy, Canadian Forest Service, advocates an approach that measures the capacity of the forest to allocate nutrients and energy in ways that increase or maintain productivity as an estimation of forest health, as a result of interaction air pollutants and climate.

Productivity in forest trees is measured by increases in radial or secondary growth. Rose-Marie Muzika, University of Missouri, used standard dendrochronological sampling methods to look at tree growth for the last 45 years and correlated her results with data for ozone, sulfur dioxide and nitrogen dioxide. Basal area increment values (BAI) were calculated and used as estimates of tree growth. She found a 50% reduction in European beech and a 20% decline for Norway spruce in her sample plots.

In related work in the U.S., McLaughlin and Downing followed seasonal growth patterns of mature loblolly pines (*Pinus taeda)* for five years. Patterns of main stem expansion and contraction, using serial measurements with sensitive dendrochnometer band systems, were recorded. Regression analyses showed that relatively low levels of ambient ozone significantly reduced growth of mature trees.

Establishing cause and effect relationships in forests, particularly in Central Europe where there are so many air pollutants, is not an easy task. Forest ecologists can use the growth analysis methods and collaborate with statisticians to attempt to account for the possible variables that affect forest health. Using tree condition alone as an endpoint does not seem to tell us enough about possible causes of the problem.

## SUGGESTED REFERENCE

McLaughlin, S.B. and Downing, D.J. (1996) Interactive effects of ambient ozone and climate measurement on growth of mature loblolly pine trees. *Can. J. For. Res.* **26**, 670.
